# Perinatal outcome of emergency cesarean section under neuraxial anesthesia versus general anesthesia: a seven-year retrospective analysis

**DOI:** 10.1186/s12871-024-02412-0

**Published:** 2024-01-19

**Authors:** Xueduo Shi, Chenyang Xu, Yazhou Wen, Ming Jiang, Huiling Yu, Xian Wang, Hongmei Yuan, Shanwu Feng

**Affiliations:** Department of Anesthesiology, Women’s Hospital of Nanjing Medical University, Nanjing Women and Children’s Healthcare Hospital, Nanjing, Jiangsu Province China

**Keywords:** Emergency cesarean section, Obstetrical anesthesia, Perinatal outcomes, Decision-to-delivery interval, Apgar score

## Abstract

**Objective:**

An emergency cesarean section (CS), which is extremely life-threatening to the mother or fetus, seems to be performed within an adequate time horizon to avoid negative fetal-maternal denouement. An effective and vigilant technique for anesthesia remains vital for emergency cesarean delivery. Therefore, this study aimed to validate the impact of various anesthesia tactics on maternal and neonatal outcomes.

**Method:**

This was a retrospective cohort study of parturient patients who were selected for emergency CS with the assistance of general or neuraxial anesthesia between January 2015 and July 2021 at our institution. The 5-min Apgar score was documented as the primary outcome. Secondary outcomes, including the 1 min Apgar score, decision-to-delivery interval (DDI), onset of anesthesia to incision interval (OAII), decision to incision interval (DII), duration of operation, length of hospitalization, height and weight of the newborn, use of vasopressors, blood loss, neonatal resuscitation rate, admission to neonatal intensive care unit (NICU), duration of NICU and complications, were also measured.

**Results:**

Of the 539 patients included in the analysis, 337 CSs were performed under general anesthesia (GA), 137 under epidural anesthesia (EA) and 65 under combined spinal-epidural anesthesia (CSEA). The Apgar scores at 1 min and 5 min in newborns receiving GA were lower than those receiving intraspinal anesthesia, and no difference was found between those receiving EA and those receiving CSEA. The DDI of parturients under GA, EA, and CSE were 7[6,7], 6[6,7], and 14[11.5,20.5], respectively. The DDI and DII of GA and EA were shorter than those of CSE, and the DDI and DII were similar between GA and EA. Compared to that in the GA group, the OAII in the intraspinal anesthesia group was significantly greater. GA administration correlated with more frequent resuscitative interventions, increased admission rates to NICU, and a greater incidence of neonatal respiratory distress syndrome (NRDS). Nevertheless, the duration of NICU stay and the incidence rates of neonatal hypoxic ischemic encephalopathy (HIE) and pneumonia did not significantly differ based on the type of anesthesia performed.

**Conclusion:**

Compared with general anesthesia, epidural anesthesia may not be associated with a negative impact on neonatal or maternal outcomes and could be utilized as an alternative to general anesthesia in our selected patient population following emergency cesarean section; In addition, a comparably short DDI was achieved for emergency cesarean delivery under epidural anesthesia when compared to general anesthesia in our study. However, the possibility that selection bias related to the retrospective study design may have influenced the results cannot be excluded.

## Introduction

As one of the commonly performed surgical procedures for parturients, emergency cesarean section (CS) is representative of the escalation of an obstetric emergency as a result of life-threatening conditions for the newborn and/or the mother [1]. Therefore, with respect to restricted time coupled with increased risk, the option of anesthesia technique is highly important for improving the fetal-maternal prognosis [2]. While GA is expected to be a widely accepted choice in urgent situations due to its advantages of rapid induction and a shortened DDI, this procedure has several underlying side effects, including failed intubation and aspiration in high-risk populations, worse umbilical arterial pH and base excess [3]. Despite the above potential risks, a retrospective survey reported the first preference for GA for emergency CS at their institution. Compared to GA, neuraxial anesthesia, recommended by the UK National Institute for Health and Clinical Excellence, has a more favorable safety profile for pregnant women indicated for emergency CS [4], with advantages covering the avoidance of potential complications, the difficulty of airway and neonatal exposure to anesthesia drugs used for intubation and maintenance of GA [5]. Therefore, regional anesthesia is increasingly the preferred anesthetic technique for pregnant women who undergo CS in emergency cases in most hospitals.

Surgical anesthesia can be established via epidural anesthesia with a well-functioning epidural catheter or rapid sequence spinal anesthesia for emergency CS, during which the onset speed of local anesthetic drugs plays an important role [6]. Remarkably, recommendations regarding the choice of local anesthetics or adjuvants with respect to the optimal type and dose for abbreviating the onset as well as potentiating high-quality anesthesia have been vague. Lignocaine (2%), 2-chloroprocaine (3%), 0.75% ropivacaine, or 0.5% bupivacaine were commonly used for cesarean delivery. The median top-up volume ranged from 16 to 19 ml for lidocaine, ropivacaine, and chloroprocaine [7]. A Bayesian network meta-analysis suggested that the onset of surgical anesthesia seemed fastest after epidural lidocaine 2% with bicarbonate, followed by 2-chloroprocaine 3% and lidocaine 2% [8]. In addition, the inclusion of adjuvants composed of opioids (fentanyl, sufentanil, and morphine), or α2-agonists (clonidine and dexmedetomidine) could result in a faster onset of anesthesia, a decreased dose of intrathecal local anesthetics and decreased occurrence of adverse events from these drugs [9]. Fentanyl combined with local anesthetics at an epidural dose of 50–75 µg or an intrathecal dose of 10–25 µg further decreased the onset time by a mean difference of more than 2 min and prolonged the postoperative analgesia duration to approximately 3–4 h [10, 11]. In addition to these advantages, α2-agonists, as additives to local anesthetics, were available for the treatment of CS patients to reduce side effects, including shivering, nausea or vomiting [11].

Although a large amount of emergency CS procedures are performed each year, to date, there is no consensus regarding the best selection of anesthesia method for emergency CS. Hence, we conducted this study to identify the influence of different types of anesthesia on maternal and neonatal outcomes and the discrepancies in DDI.

## Methods

### Study design and data sources

The study was conducted according to the principles of the Declaration of Helsinki. The study protocol was authorized by the Medical Ethics Committee of Nanjing Women and Children’s Healthcare Hospital on July 8th, 2021 (2021KY023) and was registered in the Chinese Clinical Trial Registry on August 16th, 2021 (ChiCTR2100050120). This retrospective, single-center cohort study included all patients scheduled for consecutive nonelective emergency CS from January 2015 through July 2021 and was performed at the Nanjing Women and Children’s Healthcare Hospital, a specialist maternity hospital. The data analyzed in this study were retrieved from an integrated electronic medical records system at our institution included patient hospitalization, coded diagnoses, medications, surgical and other procedures, patient characteristics, the DII, and the OAII, which was defined as the period from the end of drug injection until when the anesthesiologist would allow the surgeon to commence surgery if it was an emergency CS, DDI, and newborn or maternal condition. The 5-min Apgar score was documented as the primary outcome. The data were presented in an anonymous and standardized format.

### Participants

The inclusion criteria included patients scheduled for emergency CS, classified under ASA physical status II-V, with indications such as acute severe fetal bradycardia, placental abruption, prolapse of the umbilical cord, uterine rupture, threatened uterine rupture, eclampsia, severe hemorrhage, amniotic fluid embolism, failure of instrumental extraction with fetal distress and other life-threatening conditions for both newborns and/or mothers. Individuals with incomplete information in the electronic file and those who underwent elective operations were excluded.

### Procedures

The operating room designed for emergency CS in our obstetric delivery suite is available 24 h a day and is located just one minute away from the delivery ward equipped with monitoring facilities for both mothers and newborns. Upon receiving notification of an impending emergency, a senior obstetrician will assess whether the emergency poses a threat to the mother and/or fetus. Subsequently, the obstetrician immediately presses the emergency call button to alert the attending nurse, anesthesiologist, neonatologist, and midwife when an emergency CS is required for the parturient. An epidural top-up is administered whenever feasible using either 15 ml of 2% lidocaine or 15–20 ml of 3% chloroprocaine with or without sufentanil (20 µg) as an adjuvant. Alternatively, GA or CSEA may be performed on parturients who are contraindicated for neuraxial anesthesia or have inadequate T8 level for effective epidural labor analgesia. Parturients in the GA group received pure oxygen (100%) three minutes prior to induction of anesthesia, followed by rapid sequence induction via intravenous administration of propofol (1.5–2 mg/kg), remifentanil (1 µg/kg), and succinylcholine (2 mg/kg) to facilitate endotracheal tube insertion after loss of corneal and palpebral reflexes. After clamping of umbilical cord, midazolam (0.05 mg/kg) was administered, and maintenance of anesthesia involved continuous infusion of propofol at a concentration of 1% (80–120 µg/kg/min), sufentanil at a rate of 0.1 µg/kg/min, and cisatracurium at a rate of 2 µg/kg/min. In the CSE group, access to the epidural space was achieved using an 18G Tuohy needle at either L3-4 or L4-5 interspinous space, followed by the injection of 2 ml of 0.75% ropivacaine into the subarachnoid space through a 26G Quincke needle utilizing the needle-through-needle technique along with an epidural top-up involving the administration of 15 ml of 2% lidocaine.

### Statistical analysis

Patient characteristics were summed as descriptive statistics. The mean (standard deviation [SD]) and median (25th-75th percentile) were calculated for normally and nonnormally distributed quantitative variables, respectively. The normality of the distribution was determined using the Shapiro‒Wilk test. Normally distributed values were analyzed using variance analysis or an independent samples Student’s t test, whereas the Kruskal‒Wallis H test or Mann‒Whitney U test was used for nonnormally distributed covariates. The χ2 test or Fisher’s exact test was used to compare the differences in categorical variables. Univariable logistic regression analysis was performed for each factor, which was filtrated as candidates for multivariable regression analysis with a P value below 0.1. Multivariable regression analysis was subsequently conducted to assess the associations between the possible factors and neonatal height and weight. Missing data were handled by listwise deletion. The data analysis was conducted with IBM SPSS version 24.0. A* P* value less than 0.05 was considered statistically significant.

## Results

**Fig.1:**
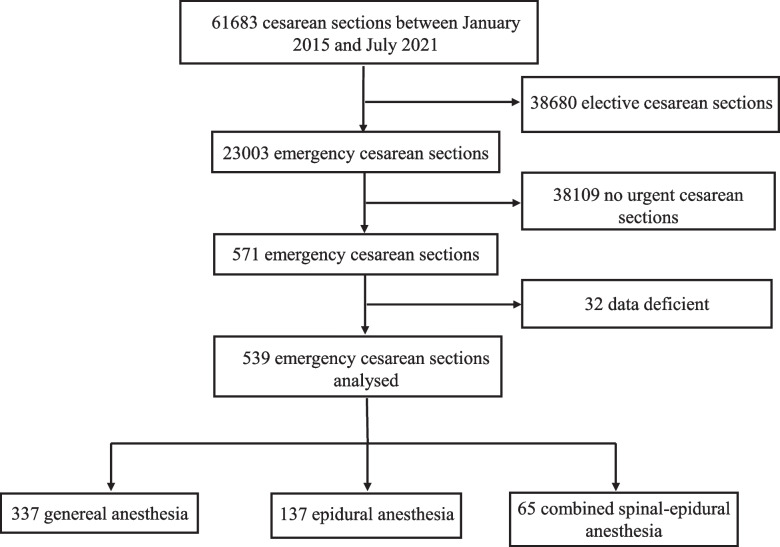
Flow chart

A total of 571 parturients underwent emergency CS between January 2015 and July 2021 at our institution, and 539 patients were eventually included in this study. GA was administered for 337 emergency CSs, 70 of whom received epidural labor analgesia before GA. EA was given to a total of 137 pregnant women, while CSEA was used for 65 individuals (Fig. 1). The characteristics of emergency CS under GA, EA, and CSEA are presented in Table 1.

**Table 1:** Characteristics of parturients underwent emergency cesarean section under general or neuraxial anesthesia

Variables	GA group	EA group	CSE group	*P*
Age, year	32.0 ± 4.2	31.4 ± 3.5	32.1 ± 4.0	*P* > 0.05
BMI, kg.m^−2^	26.56 ± 3.35	26.05 ± 2.67	26.78 ± 3.35	*P* > 0.05
ASA, n				*P* < 0.05
II	278(82.5%)	127(92.7%)	59(90.8%)	
III	55(16.3%)	10(7.3%)	6(9.2%)	
IV	2(0.6%)	0	0	
V	2(0.6%)	0	0	
Gestational age, week	39.4[37.9,40.4]	39.9[39.1,40.6]	39.0[37.8,40.1]	*P* < 0.0001
Primipara, n				*P* < 0.0001
Yes	288(85.5%)	124(90.5%)	15(23.1%)	
No	49(14.5%)	13(9.5%)	50(76.9%)	
Indications for urgent CS, n				
Acute severe fetal bradycardia	282(83.7%)	130(94.9%)	30(46.2%)	*P* < 0.0001
Placental abruption	10(3.0%)	0	7(10.8%)	*P* < 0.0001
Prolapse of umbilical cord	21(6.2%)	4(2.9%)	0	*P* > 0.05
Uterine rupture	1(0.3%)	0	0	*P* > 0.05
Eclampsia	5(1.5%)	1(0.7%)	1(1.5%)	*P* > 0.05
Other	17(5.0%)	2(1.5%)	26(40%)	*P* < 0.0001
Preexisting labor analgesia, n				*P* < 0.0001
Yes	70(20.8%)	92(67.2%)	2(3.2%)	
No	267(79.2%)	45(32.8%)	63(96.9%)	
Duration of operation, min	32[27,37]^a^	31[26,35]	28[25,33]	0.004

The data were presented as mean ± standard deviation (Mean ± SD) or median [P25, P75] or number (percentage); *GA* general anesthesia, *EA* epidural anesthesia, *CSE* combined spinal-epidural anesthesia, *BMI* Body Mass Index, *ASA* American Society of Anesthesiologist; Preexisting labor analgesia: Epidural labor analgesia was performed prior to emergency CS

^a^*p* < 0.05 in comparison with CSE group

The Apgar scores at the first and fifth minutes were lower in GA group than those in the EA and CSE groups. The percentage of patients with an Apgar score < 7 at one minute was recorded as 10.4% under GA, whereas it was only 0.7% for EA and 1.5% for CSEA. There was no significant difference concerning the incidence of Apgar score < 7 at five minutes among three groups (*P* > 0.05). No statistically significant difference was observed between the EA and CSE groups concerning a two-point decrease in the Apgar score (*P* > 0.05). The rate of Apgar score < 3 at both one and five minutes in GA group did not statistically differ from that in the neuraxial groups (*P* > 0.05). GA administration correlated with more frequent resuscitative interventions, increased admission rates to NICU, and a greater incidence of NRDS in our analyzed patients. Nevertheless, the duration of NICU stay and the incidence rates of HIE and pneumonia did not significantly differ based on the type of anesthesia performed. No significant difference was detected regarding birth height or weight through multivariate logistic regression analysis, although the two indices were statistically lower in newborns who underwent GA (Tables 2 and 3).

**Table 2:** Outcomes of neonatus underwent emergency cesarean section under general or neuraxial anesthesia

Variables	GA group	EA group	CSE group	*P* value
Apgar score				
1 min	10[8,10]^a,b^	10[10,10]	10[10,10]	*P* < 0.0001
5 min	10[10,10]^a,b^	10[10,10]	10[10,10]	*P* < 0.0001
Apgar score < 7				
1 min	35(10.4%)^a^	1(0.7%)	1(1.5%)	*P* < 0.0001
5 min	9(2.7%)	0	1(1.5%)	0.111
Apgar score < 3				
1 min	10(3.0%)	0	1(1.5%)	0.108
5 min	3(0.9%)	0	0	0.701
Birth height, cm	48.90 ± 3.09^a^	49.88 ± 1.21	49.32 ± 1.87	0.001
Birth weight, g	3062.72 ± 652.90^a^	3271.17 ± 397.70	3169.54 ± 515.82	0.002
Resuscitation, n	37(11.0%)^a^	3(2.2%)	5(7.7%)	0.003
Admission to NICU, n	78(23.1%)^a^	4(2.9%)^b^	10(15.4%)	*P* < 0.0001
Duration of NICU,day	6[4, 13]	4.5[4, 6.5]	6[4.5, 8.5]	0.469
Complications, n				
NRDS	22(6.5%)^a^	1(0.7%)	2(3.1%)	0.01
NHIE	5(1.5%)	1(0.7%)	2(3.1%)	0.352
PDA	20(5.9%)	2(1.5%)	2(3.1%)	0.085
PFO	34(10.1%)^a^	3(2.2%)	2(3.1%)	0.003
Pneumonia	16(4.7%)	1(0.7%)	3(4.6%)	0.071
Child death, n	3(0.9%)	0	0	0.701

Data were expressed as mean ± standard deviation (Mean ± SD) or median [P25, P75] or number (percentage); *NRDS* Neonatal Respiratory Distress Syndrome, *NHIE* Neonatal Hypoxic Ischemic Encephalopathy, *PDA* Patent Ductus Arteriosus, *PFO* Patent Foramen Ovale

^a^*p* < 0.05 in comparison with EA group

^b^*p* < 0.05 in comparison with CSE group

**Table 3:** Multivariate logistic regression analysis to factors of the height and weight of neonatus

Variables	Height			Weight		
	β	t	*P* value	β	t	*P* value
BMI, kg.m^−2^	0.136	3.717	< 0.001	0.051	1.413	< 0.001
Gestational age, week	0.478	12.851	< 0.001	0.494	13.356	< 0.001
Type of anesthesia						
GA	-0.070	-1.243	0.214	-0.060	-1.057	0.291
EA	0.063	1.520	0.129	0.082	2.161	0.31
CSEA	0.040	1.044	0.297	0.023	0.625	0.532
Labor analgesia before GA	0.056	1.332	0.183	0.053	1.253	0.211

*P* < 0.05 was considered statistically significant

The overall median DDI was reported as 7 [6, 7] min. A DDI ≤ 5 min occurred in 91 (16.9%) women following emergency CS, and for 357(66.2%) parturients, the DDI ranged from 5 to 10 min. A median DDI of 6 [6, 7] was recorded for subjects following the anesthetic method of EA, and 26 (19.0%) of those clients had DDIs less than 5 min. Group EA exhibited a parallelly short DDI and DII intervals compared to the group GA (*P* > 0.05). Compared to those of subjects in the GA or EA group, the DDI of 14 [11.5, 20.5] combined with the DII of the CSE group were significantly greater (*P* < 0.05) (Table 4).

**Table 4:** Intraoperative outcomes of parturients underwent emergency cesarean section under general or neuraxial anesthesia

Variables	GA group	EA group	CSE group	*P* value
DDI, min	7[6,7]^b^	6[6,7]^b^	14[11.5,20.5]	*P* < 0.0001
DDI ≤ 5, n(%)	65(19.3%)	26(19.0%)	0	
5 < DDI ≤ 10, n(%)	253(75.1%)	104(75.9%)	14(20.9%)	
10 < DDI ≤ 15, n(%)	14(4.1%)	3(2.2%)	23(34.3%)	
15 < DDI ≤ 20, n(%)	3(0.9%)	4((2.9%)	12(17.9%)	
20 < DDI ≤ 30, n(%)	2(0.6%)	0	15(22.4%)	
DDI > 30, n(%)	0	0	1(1.5%)	
DII, min	5[4,5]^b^	5[4,5.5]^b^	12[8, 17]	*P* < 0.0001
OAII, min	1[1,2]^a,b^	2[1,3]^b^	5[3, 8]	*P* < 0.0001
Blood loss, mL	400[380,565]^b^	400[400,500]^b^	390[350,500]	*P* < 0.0001
Transfusion, n (%)	14(4.2%)^a^	0	0	0.01
Vasoactive drug, n (%)	12(3.6%)	5(3.6%)	6(9.2%)	0.108
Hospitalization, day	6[5,7]^b^	6[6,7]^b^	6[5,6.5]	0.001

Data were expressed as median [P25, P75] or number (percentage); *DDI* Decision to delivery interval, *DII* Decision to incision interval, *OAII* Onset of anesthesia to incision interval

^a^*p* < 0.05 in comparison with EA group

^b^*p* < 0.05 in comparison with CSE group

For all obstetric patients in the GA group, labor epidural analgesia was performed for 70 parturients who underwent emergency CS before receiving general anesthesia. Compared to that of patients without labor epidural analgesia before the induction of GA, the DII of patients receiving preexisting labor epidural analgesia was lower combined with increased Apgar score of 5th min (*P* < 0.05). No significant association was found between labor analgesia and birth weight according to multivariate analysis (Table 3). There were no significant differences in terms of DDI, OAII, duration of surgery, birth height, blood loss, or hospitalization (*P* > 0.05) (Table 5).

**Table 5:** Data of parturients according to labor epidural analgesia before emergency CS under general anesthesia

Variables	LEA group	Without LEA group	*P* Value
DDI, min	6[5, 7]	7[6, 7]	0.059
DII, min	5[4, 5]	5[4, 6]	0.022*
OAII, min	1[1, 2]	1[1, 2]	0.156
Duration of surgery, min	32[26,38.5]	32[27,37]	0.807
Apgar score			
1 min	10[9, 10]	10[8, 10]	0.142
5 min	10[10, 10]	10[10, 10]	*P* < 0.0001
Birth height, cm	50[50,50]	50[49,50]	0.153
Birth weight, g	3280[2910,3565]	3180[2777.5,3452.5]	0.043*
Blood loss, mL	400[375,562]	400[380,580]	0.932
Hospitalization, day	6[6, 7]	6[5, 7]	0.348

Data were presented as median [P25, P75]; *LEA* Labor epidural analgesia, *DDI* decision to delivery interval, *DII* decision to incision interval, *OAII* onset of anesthesia to incision interval

^*^P < 0.05

## Discussion

As one of the most commonly performed surgeries worldwide, cesarean section is one mode of labor for decreasing maternal and perinatal morbidity and mortality [12]. Hence, more attention should be given to the effect of anesthetic patterns on perinatal outcomes. In recent years, with the maturity of neuraxial anesthesia coupled with the improved safety of general anesthesia, there has been a reduction in anesthesia-associated obstetric mortality [13]. In this retrospective study, we demonstrated that epidural anesthesia had comparable potential relative to general anesthesia in terms of DDI, DII, and material outcomes, including blood loss, vasoactive drugs, and hospitalization. Moreover, the higher Apgar scores of the 1st and 5th min as well as lower admission to NICU of newborns were observed in newborns who received epidural anesthesia in comparison with those who received general anesthesia in our selected cases. In addition, the DDI in our institution was limited to within 30 min for nearly all obstetrics, with a rate of 86.2% for a DDI less than 10 min for GA and EA. Notably, the retrospective design limits the interpretation of the results without full consideration of selection bias.

Guaranteeing security for pregnancy following CS in emergent cases remains a challenge for anesthesiologists. Typically, the indications for the type of anesthetic technique depend on the degree of urgency in relation to maternal and fetal status and comorbidities as well as on the difficulty or expected duration of procedures [5]. Although general anesthesia is regarded as a generally accepted choice in emergent situations due to its rapid and predictable onset, the procedure has several underlying side effects [14]. Notably, all obstetric patients are at a high risk for pulmonary aspiration when they are receiving general anesthesia [15], suggesting an eightfold-fold higher risk than that of non-obstetrical patients with respect to failed intubation and aspiration [16]. An investigation by Kinsella et al. revealed that the incidence of obstetric failed tracheal intubation remained stable at 2.3 per 1000 GA for CS, and maternal mortality from failed intubation was 2.3 per 100,000 GA, while aspiration or hypoxemia was secondary to airway obstruction or esophageal intubation [17]. In addition, pregnant women diagnosed with severe preeclampsia and undergoing emergency CS are prone to stroke due to high incremental blood pressure and neuroendocrine stress responses without the addition of opioids to execute GA [18]. A retrospective cohort analysis of 194 code-red cesarean sections conducted by Cyril et al. verified the close relationship between GA and negative well-being of newborns [19]. Algert et al. reported that among the infants who required intubation, those delivered via GA had a 5-min Apgar score of < 7, which was more common than that of infants delivered via regional anesthesia [20]. In our study, babies delivered under GA had decreased Apgar scores at both 1 and 5 min compared with those who received intraspinal anesthesia, which was analogous to the findings of previous studies. Besides, more frequent resuscitations and transfers to the NICU were observed in patients who received GA in our cohort. A possible explanation we postulated, as mentioned in previous studies, is that general anesthesia may affect neonatal conditions to some extent because of transient sedation of the neonate from the anesthetic drugs [19]. However, a causal relationship could not be drawn in our study considering the small sample size and the retrospective design.

The preponderance of neuraxial anesthesia, as the preferred anesthetic technique for cesarean section in cases of emergent situations by anesthetists, is composed of the avoidance of the potential complications of difficult airways, aspiration of gastric content, neonatal exposure to anesthetic drugs applied during the period of anesthetic induction and maintenance of GA [5, 14], and the requirement of a low dose and concentration of local anesthetics [2]. Hence, some scholars have proposed that regional anesthesia should be executed whenever possible, as it was associated with shorter hospital stays, less maternal morbidity, and higher Apgar scores and umbilical blood pH values in neonates [2, 20]. In additions, the conversion of epidural analgesia to surgical anesthesia for emergency cesarean delivery in parturients with effective labor epidural catheter was not associated with poorer outcomes in newborns [21]. In this retrospective study, a lower incidence of Apgar scores < 3 for infants delivered via EA or CSEA was recorded, and the hospitalization of patients in the CSE group was shorter than that of patients in the GA or EA group.

The DDI was defined as the time taken from recognition of an abnormality on fetal heart tracing using cardiotocography and decision to proceed with operative delivery to the time of delivery of the fetus. Until now, no consensus has been reached concerning the ideal DDI, a quality indicator of emergency CS, or its influence on maternal outcome and neonatal well-being [22]. A time recommendation limiting DDI to 30 min for emergency CS procedure has been advocated by the Royal College of Obstetricians and Gynecologists as well as the American College of Obstetricians and Gynecologists [23]. However, the incidence of DDI ≤ 30 min was reported to be only 17.5% in a retrospective cross-sectional study of 510 mothers who underwent emergency CS [24]. A prospective study analyzing 163 category-1 emergency cesarean sections reported that the average DDI was 42 ± 21.4 min, with a prevalence of only 19.6% of women having a DDI below 30 min [25]. In our unit, delivery could be achieved within the recommended time interval, during which the DDI within 10 min was nearly 86.2% of the parturients combined with the rate of 93.7% for DDI ≤ 15 min. In addition, compared to patients without labor epidural analgesia before the induction of GA, no significant difference was found considering DDI for patients administered labor epidural analgesia beforehand. The DII of the GA group receiving preexisting labor epidural analgesia was lower than that without labor epidural analgesia before GA, which was ascribed to the effect of labor epidural analgesia to some extent.

In addition, we found similarly short DDI intervals between the GA and EA groups, differing from several lines of evidence indicating that the technique of regional anesthesia was associated with prolonged DDI compared to general anesthesia [26]. The epidural top-up through an epidural catheter already inserted and providing effective analgesia might be the predominant factor responsible for shortening the time interval of epidural anesthesia [19, 27]. Another possible reason might be that the administration of chloroprocaine accelerated the onset of EA. In our unit, we also noted that performing CSEA was more time-consuming than performing GA or EA, as verified by the prolonged interval of DDI. However, the sample of CSEA patients in our study was so small that further study is warranted to verify the reliability of the results.

There are some limitations in the present study. First, our study was a single-center retrospective analysis; therefore, confounding effects and bias are inevitable to some extent. Second, the present results should not be extrapolated to other surgical types considering the single-center nature of the study with small sample sizes and the retrospective design, and further investigations are warranted to confirm the results. Third, we did not record the umbilical blood pH values, which reflect neonatal outcomes. Finally, long-term outcomes were not measured in our study.

## Conclusion

Epidural anesthesia may not be associated with a negative impact on neonatal and maternal outcomes compared to general anesthesia and could be utilized as an alternative to general anesthesia in our selected patient population following emergency cesarean section; in addition, comparably short DDI was achieved for emergency cesarean delivery under epidural anesthesia when compared to general anesthesia in our study.

## Data Availability

The datasets generated and/or analyzed during the current study are not publicly available due to limitations of ethical approval involving the patient data and anonymity but are available from the corresponding author on reasonable request.
